# Mitochondrial Deoxyribonucleic Acid Copy Number Elevation As a Predictor for Extended Survival and Favorable Outcomes in High-Grade Brain Tumor Patients: A Malaysian Study

**DOI:** 10.5152/eurasianjmed.2024.23172

**Published:** 2024-02-01

**Authors:** Siti Muslihah Ab Radzak, Siti Zulaikha Nashwa Mohd Khair, Zamzuri Idris, Wan Muhamad Amir Wan Ahmad, Azim Patar, Abdul Aziz Mohamed Yusoff

**Affiliations:** 1 Department of Neurosciences, Universiti Sains Malaysia School of Medical Sciences, Kubang Kerian, Kelantan, Malaysia; 2 Department of Biostatistics, Universiti Sains Malaysia School of Dental Sciences, Kelantan, Malaysia, Kelantan, Malaysia

**Keywords:** mtDNA copy number, brain tumors, survival predictor

## Abstract

**Background:**

Investigating the role of mitochondrial DNA (mtDNA) alterations and their impact on brain tumor progression remains a significant focus in cancer research. The research aimed to explore the specific contributions of mtDNA copy number changes and their correlations with patient survival, large mtDNA deletions, and *TFAM* mutations in brain tumor patients.

**Methods:**

A total of 41 patients with confirmed brain tumors underwent DNA extraction from both tumor tissues and blood samples. The relative mtDNA copy number in comparison to the nuclear genome was quantified using quantitative real-time polymerase chain reaction (qRT-PCR). Long-range PCR assessed large-scale mtDNA deletions, and Sanger sequencing was applied to detect exon 4 *TFAM* mutations.

**Results:**

Analysis revealed significantly increased mtDNA copy numbers in brain tumor tissues (80.5%) compared to matched blood samples (*P* < .001). Median delta Ct (∆Ct) values were 7.35 in cancerous tissues and 11.81 in blood (*P* < .001), with median relative mtDNA content of 0.0123 and 0.0006, respectively (*P* < .001). Patients with higher mtDNA copy numbers experienced longer overall survival periods (*P* = .045) and notably favorable outcomes, particularly in high-grade tumor cases (*P* = .016). Furthermore, a single-nucleotide deletion was identified in exon 4 of *TFAM* in a patient with glioblastoma IV, while no large-scale mtDNA deletions were found in brain tumor patients.

**Conclusion:**

Our study strongly supports the role of increased mtDNA copy numbers as a reliable predictor for improved survival and positive outcomes in high-grade brain tumor patients.

**Main Points**

- Brain tumors are heterogeneous diseases, and the molecular landscape is complex and complicated.
- Diverse molecular alterations in mitochondrial DNA (mtDNA), including point mutations, deletions, insertions, microsatellite instability, polymorphisms, and changes in mtDNA copy number, have been identified and characterized in human brain tumors.
- Brain tumor screening in Malaysian patients reveals an increase in mtDNA copy number.
- The association of the increased mtDNA copy number and the clinicopathological features of brain tumor patients show no significant differences.
- The elevated mtDNA copy number shows higher overall survival in patients and a better prognosis in the high-grade tumor group.

## Introduction

Brain tumors rank among the deadliest human malignancies, causing substantial morbidity and mortality worldwide. The Global Cancer Observatory report estimated a global incidence of 308 102 new cases and 251 239 deaths from brain tumors in 2020.^1^

Various cellular and molecular research methods highlight the molecular diversity of brain tumors, arising from the progressive gathering of genetic mutations and epigenetic alterations.^2^ Yet, comprehending the origins of both malignant and non-malignant brain tumors, especially regarding the genetic aspects of the mitochondrial genome, remains a challenging endeavor.

Renowned for their vital involvement in cellular energy production and contribution to reactive oxygen species (ROS), mitochondria play a crucial role in maintaining metabolic homeostasis and regulating the process of cell death. Possessing their own genome, mitochondria are believed to have evolved independently from nuclear DNA. The suspicion of mitochondrial dysfunctions playing a role in boosting tumor growth and facilitating cancer progression has persisted for an extended period.

It is widely acknowledged that cells typically house hundreds to thousands of copies of mtDNA, highlighting its abundance within cells. The regulation of mtDNA copy number is influenced by cellular physiological states and can vary due to factors like aging, hormonal treatments, cellular differentiation, and physical activity.^3^ Nevertheless, detailed insights into the mechanisms governing abnormal mtDNA content in cancer progression are currently limited and lack clarity. Alterations in mtDNA copy number are thought to play a role in fostering genomic instability, thereby enhancing the advancement of tumors. Up to this point, both an augmentation and reduction in mtDNA copy number have been identified as potential risk factors for cancer in human studies.^4^

Significantly, the regulation of mtDNA replication influences the quantity of mtDNA copies, a process mediated by distinct enzymes and proteins, such as mitochondrial transcription factor A (*TFAM*). *TFAM*, a transcription factor encoded in the nucleus and functioning within mitochondria, holds a pivotal position in both mtDNA replication and the intricate process of mitochondrial biogenesis.^5^ Supported findings suggest that *TFAM* has a strong correlation with the abundance of mtDNA copies and plays a crucial role in regulating mtDNA expression.^5^ Cells depleted of mtDNA exhibit diminished mitochondrial function, lowering *TFAM* protein expression in both the liver and skeletal muscle.^6^ Furthermore, cells with *TFAM* knockdown demonstrate a decrease in mtDNA copy numbers, as observed in esophageal squamous cell carcinoma.^7^

Alterations or deletions within mtDNA sequences have the potential to induce irregularities in mitochondrial copy numbers. In these scenarios, defects in mtDNA can lead to deletions by causing double-strand DNA breakage.^8^ Significantly, large-scale mtDNA deletions, often associated with mtDNA content levels, have been commonly observed in the aging process and mitochondrial myopathies and have recently been revealed in various human cancers.^9,10^

In the present study, we assessed the alterations that occur in the mtDNA genome and the role of mtDNA content in brain tumor patients. The implications of the aberrant mtDNA copy number in brain tumors demand novel observations, specifically in distinct tumor types and grades. Hence, this research aimed to investigate alterations in mtDNA and variations in copy numbers among Malaysian patients diagnosed with brain tumors, aiming to explore their connections with survival rates and other clinical characteristics.

## Material and Methods

### Case Population

The study involved 41 brain tumor tissues obtained from the Department of Neurosciences, Universiti Sains Malaysia, between 2019 and 2023. To serve as a comparison, peripheral blood samples were collected from the same patients, serving as the control group. These samples specifically comprised histologically confirmed primary brain tumors, classified by neuropathologists following World Health Organization (WHO) guidelines. Additionally, individuals who had undergone prior neoadjuvant therapy and cases of tumor recurrence were excluded from this study. This study followed the principles outlined in the Declaration of Helsinki, and written informed consent was obtained from all participants. Ethical approval for the study was granted by the Research Ethics Committee of Universiti Sains Malaysia (approval no: USM/JEPem/17050269, Date: 26th July 2019).

### *DNA *Extraction

DNA extraction was carried out using the Geneaid Isolation Kit (Geneaid Biotech Ltd., New Taipei City, Taiwan) following the manufacturer’s protocols. The concentration and quality of the isolated DNA were assessed using the NanoDrop ND1000 spectrophotometer and 1% gel agarose electrophoresis. All eligible DNA samples were preserved at −80°C until further analysis.

### *mtDNA *Content Analysis

The relative mtDNA copy number was assessed via quantitative polymerase chain reaction (qPCR) by concurrently amplifying mtDNA (*ND1 *gene) and nuclear DNA (*β-actin *gene) using 2× Brilliant III SYBR Green Master Mix with Low Rox on the Mx3005P Real-time PCR System (Agilent Technologies, Inc., Santa Clara, Calif, USA) for all available samples. Primers for *ND1* were 5’-TCTCACCATCGCTCTTCTAC-3’ as the forward primer and 5’-TTGGTCTCTGCTAGTGTGGA-3’ as the reverse primer. For the *β-actin* gene, 5’-CATGTGCAAGGCCGGCTTCG-3’ was used as the forward primer and 5’-CTGGGTCATCTTCTCGCGGT-3’ as the reverse primer. The cycling conditions were 95°C for 3 minutes, followed by 40 cycles of 15 seconds at 95°C and 20 seconds at 60°C. The samples were run in triplicates, and the average threshold cycle number (Ct) values for both genes were used to determine the relative mtDNA content. The determination of mtDNA content was measured using the formula 2 × 2^−∆CT (CtmtDNA – CtnDNA)^ and 2^−∆∆CT^ method.

### Long-Range Polymerase Chain Reaction for Large-Scale mtDNA Deletions Analysis

The process of detecting mtDNA large-scale deletions was conducted following similar protocols used previously, albeit with slight modifications.^10^ Two divided fragments of the entire mtDNA, which are 7.8 kb and 9.3 kb, were performed by long-range PCR. For the 7.8 kb amplification, 30 cycles were used with 98°C for 10 seconds, 68°C for 30 seconds, and 72°C for 3 minutes 30 seconds, and a final elongation at 72°C for 10 minutes. For the amplification of the 9.3 kb, the cycling conditions used were 30 cycles of 98°C for 10 seconds, 68°C for 30 seconds, and 72°C for 5 minutes, with a final elongation at 72°C for 10 minutes. The reaction mixture used for both amplifications was Phusion High Fidelity (ThermoFisher Scientific, Waltham, Mass, USA) on the SureCycler 8800 Thermal Cycler (Agilent Technologies, Inc., Santa Clara, Calif, USA). The products were then checked on a 1% agarose gel in TAE buffer for 55 minutes at 75 volts.

### TFAM Mutation Analysis

The amplification of exon 4 of *TFAM* was performed as stated previously.^2^ The PCR was conducted using the SureCycler 8800 Thermal Cycler (Agilent Technologies, Inc., Santa Clara, Calif, USA). A volume of 20 µL total reaction was performed using Phusion High Fidelity (ThermoFisher Scientific) for 30 cycles of 98°C for 10 seconds, 68°C for 30 seconds, and 72°C for 30 seconds, and a final elongation at 72°C for 7 minutes. The PCR products were then purified using QIAamp Purification Kit (QIAGEN, Hilden, Germany).

### Sanger Sequencing

The PCR products were purified and subjected to Sanger sequencing using the BigDye® Terminator v3.1 cycle sequencing kit (Applied Biosystems, Foster City, Calif, USA) on an ABI Prism 3700 DNA Analyzer automated sequencer (Applied Biosystems, Foster City, Calif, USA). The sequencing of the purified PCR products was executed with the same primers used in the PCR amplification process. Electropherogram results were aligned using BLAST software from the NCBI site, and the complementary DNA sequences for the *TFAM* gene (NC_000010.11) available in the database were used as references.

### Statistical Analysis

The statistical analysis was conducted using Statistical Package for the Social Sciences (SPSS), version 28 (IBM SPSS Corp.; Armonk, NY, USA). The mtDNA content values are presented as the median, the interquartile range (IQR), and the fold difference. The Mann–Whitney *U-*test was applied to analyze the relationship between mtDNA content and clinicopathological features among brain tumor patients, and the Pearson correlation coefficient was used to determine the relationship of the mtDNA content between cancerous tissues and blood controls. The chi-square difference test was used to compare the differences between the case groups. Kaplan–Meier survival analysis, using the log-rank test to determine significance, was performed to evaluate the survival of patients based on variations in mitochondrial DNA copy numbers. Survival duration was measured from the initial surgery day to the last clinical follow-up or relapse occurrence. The results were considered statistically significant at a *P*-value less than .05.

## Results

### Clinicopathological Features

The clinicopathological characteristics of the patients are summarized in Table 1. A total of 41 patients with brain tumors were enrolled in this study, 23 (56.1%) were males, and 18 (43.9%) were females, with their age at diagnosis ranging from 5 to 73 years (mean: 41.9 ± 18.63). According to WHO classification, these brain tumors were categorized into 2 groups, glial (58.5%) and non-glial (41.5%) cases, and 2 distinct tumor grades, low grade (I and II; 61.0%) and high grade (III and IV; 39.0%) tumors. The highest number of cases was represented by 26.8% (11/41) meningioma I and followed by 12.2% (5/41) schwannoma I and glioblastoma multiforme IV. Each group of oligodendrogliomas III and anaplastic pleomorphic xanthoastrocytomas III displayed 9.8% (4/41) of the cases, while each group of gemistocytic astrocytoma II, ependymoma II, medulloblastoma IV, and meningioma II constitute 7.3% (3/41) of all cases.

### Determination of mtDNA Content in Co-extracted Samples

Total DNA of mtDNA and nuclear DNA were co-extracted from paired cancerous tissues and blood samples. The results revealed that the average Ct values for the *MTND1* gene, which constitutes total mtDNA, ranged from 22.05 to 32.38 in cancerous brain tissues and from 27.13 to 34.74 in peripheral blood, respectively. The average Ct values of the *ACTB* sequence, representing nDNA, ranged from 16.69 to 22.67 in cancerous tissues and from 17.86 to 23.22 in blood controls, respectively. The average Ct values of *ACTB* were less compared to the average Ct values of *MTND1*, indicating a greater level of nDNA than that of mtDNA level in all cases of brain tumors.

**Figure 1.**
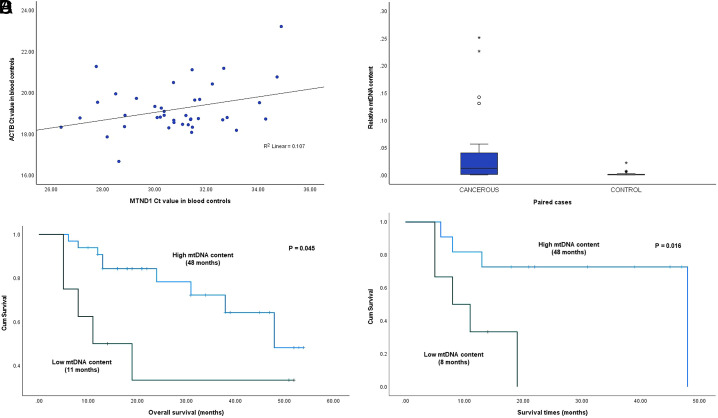
A. mtDNA amplifications are correlated with nDNA amplifications in blood controls (*P* = .039, *r* = 0.327). B.Comparison of mtDNA content in cancerous brain tissues and blood controls. C. The overall survival plot of brain tumor patients with high and low mtDNA copy number. D. The survival plot of high-grade brain tumor patients with high and low mtDNA copy number.

In this study, we found no correlation between the average Ct values of *MTND1* amplification with the *ACTB* amplification in cancerous tissues (*r* = 0.185; *P* = .248). Conversely, a significant correlation of both amplifications was observed in blood controls (*r* = 0.327; *P* = .039) (Figure 1A).

### mtDNA Copy Number in Cancerous Tissues and Paired Blood Specimens

The present findings demonstrated that mtDNA content was significantly increased in cancerous brain tumor tissues (33/41) than those in the blood controls (chi-square difference test, *P* < .001) (Figure 1B). The ∆Ct values between cancerous tissues and blood controls showed a 4.5 cycle difference (∆∆CT) (Mann–Whitney *U*-test, *P* < .001), and the fold change of mtDNA content (2^−∆∆CT^) was 22.6 cycles. Additionally, there was a marked difference between relative mtDNA content in cancerous tissues and blood controls (2 × 2^−∆CT^) (Mann–Whitney *U*-test, *P* < .001) (Table 2).

### Associations of Increased mtDNA Content and Clinicopathological Features in Brain Tumors

The associations between the relative content of mtDNA and other clinical parameters of 41 brain tumor cases, including gender, age at the time of diagnosis, tumor grades, and histological tumor types, were analyzed (Table 3). At the *α* = 0.05 level of significance, the outcomes show [median_male_ (IQR) = 0.0063 (0.03), median_female_ (IQR) = 0.0186 (0.04); *Z* = 240.5; *P *> .05] that there was no significant difference in the median score (*P* = .379) between males and females. Similarly, the results for the age group also showed no significant difference (*P* = .331) [median_<40_ (IQR) = 0.0115 (0.04), median_≥ 40_ (IQR) = 0.0123 (0.04); *Z* = 244.0; *P *> .05].

Moreover, the data uncovered that the groups for tumor grades and histological types of brain tumor patients were not significantly different when *P*= .570 [median_low _(IQR) = 0.0154 (0.04), median_high_ (IQR) = 0.0078 (0.05); *Z* = 178.5; *P *> .05] and *P* = .534 Median_glial _(IQR) = 0.0087 (0.03), Median_non-glial_ (IQR) = 0.0203 (0.04); *Z* = 227.5; *P *> .05], respectively. In conclusion, the median score of gender, age, tumor types, and histological types of brain tumors did not significantly differ between the groups.

### Elevated mtDNA Copy Numbers Correlate with Extended Overall Survival in Patients with Brain Tumors

In this study, Kaplan–Meier survival analysis was used to compare the overall survival rates between 2 groups based on mtDNA copy number (Figure 1C). The results indicated that patients in the increased mtDNA copy number group showed an average survival of 48 months, whereas those in the decreased mtDNA copy number group had an average survival of approximately 11 months (*P* = .045). This finding suggests that individuals in the increased mtDNA copy number group experienced significantly longer survival durations in comparison to those in the decreased mtDNA copy number group.

Furthermore, an examination was conducted to analyze the survival outcome concerning the groups categorized by mtDNA copy number and clinical variables. The analysis revealed that high-grade tumor patients with increased mtDNA copy numbers exhibited better survival rates compared to those in the decreased mtDNA copy number group (*P* = .016) (Figure 1D). However, no significant impact on the survival times of brain tumor patients was found in relation to other clinical parameters such as age, gender, and histological tumor types.

### Large-Scale mtDNA Deletion Analysis

**Figure 2.**
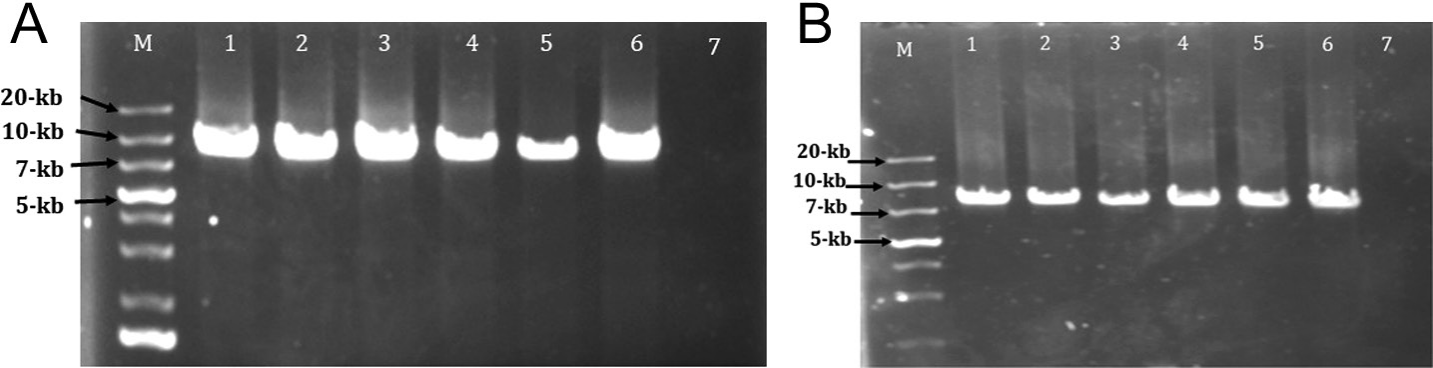
A. The mtDNA genome of the samples was amplified in 9.3 kb, implying the absence of large-scale mtDNA deletions. B. The mtDNA genome of the samples was amplified in 7.8 kb, implying the absence of large-scale mtDNA deletions in brain tumors. Lane M, marker; lane 1-3, cancerous tissues; lane 4-6, blood controls; lane 7, no-template control to monitor contamination.

Large-scale deletions of the mtDNA genome were considered in the brain tumor patients based on the absence of amplification PCR products at 7.8 kb and 9.3 kb (Figures 2A and B). The presence of these 2 amplicons indicated no large-scale mtDNA deletions in the samples. For 41 of the samples examined, the long-range PCR assay failed to detect samples that harbored large-scale mtDNA deletions.

### *TFAM *Mutation

**Figure 3.**
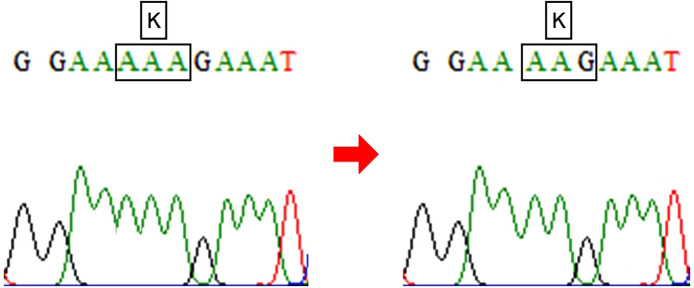
Detection of a single deletion of nucleotide A (Lys3360) in exon 4 of *TFAM *sequences by direct sequencing.

A fragment of exon 4 of *TFAM* was successfully amplified by specific primers, indicating a single PCR amplicon with the desired product at 200 bp in size. In the present study, *TFAM* mutation analysis uncovered that only 1 patient (1/41) exhibited nucleotide changes in patients who suffered from GBM IV. According to the electropherogram data, a single deletion of nucleotide A at position 3360 (Lys3360) caused no change in the amino acid sequence was observed (Figure 3).

## Discussion

The prevailing consensus acknowledges that changes in mtDNA copy number and malfunctioning mitochondria play crucial roles in cancer advancement, extensively studied across various research.^5,11^ Despite adequate evidence connecting irregular mtDNA levels to tumor development, the origins and underlying understanding of this anomaly remain uncertain. Therefore, this study aimed to evaluate the functions of altered mtDNA copies, integrating mutation analyses, potentially influencing the invasiveness of brain tumors.

In this study, we assessed the mtDNA copy number in brain tumor tissues and blood samples. The findings revealed that the relative mtDNA content was significantly elevated in 33 out of 41 (80.5%) brain tumor tissues compared to their corresponding blood controls. As far as we know, our recent findings represent the initial observation of mtDNA content across various types and grades of brain tumors, encompassing gliomas, meningiomas, schwannomas, and medulloblastomas. Previous studies have presented inconclusive and conflicting outcomes regarding mtDNA copy numbers, underscoring the intricacies associated with mtDNA copies in brain tumor cases.^12-15^ The summary of previous reports of mtDNA content variations in most glioma cases has been documented in Table 4.

Additionally, our findings align with earlier research that noted a greater ratio of mtDNA content in tumor tissues compared to non-tumor tissues in GBM patients.^16^ Correspondingly, research conducted by Zhang et al. revealed heightened mtDNA copy numbers in glioma patients’ tumor cases compared to controls, strongly linked to glioma susceptibility.^17^ In contrast, studies by Shen H et al^18^ and Shen J et al^19^ reported a decreased mtDNA copy number in glioma tissues compared to those in corresponding non-tumorous specimens.

The present study demonstrated elevated mtDNA copy numbers per cell ranging from 2^-2.99^ to 2^-13.84^ in tumorous tissues compared to those in peripheral blood controls, which ranged from 2^-6.48^ to 2^-15.57^. Additionally, the average mtDNA content between brain tumorous tissues and blood controls appeared to have statistically significant differences. These outcomes indicate the enhanced sensitivity of the measurement of mtDNA alterations in cancerous tissues compared to blood specimens, providing a reassuring clinical appraisal of tumor progression. A comparable finding was reported when assessing mtDNA copy numbers in exosomes derived from plasma and brain tissue of glioblastoma (GBM) patients. The researchers observed reduced mtDNA content in both exosomes and brain tissues of tumor samples compared to the control group, suggesting that exosome analysis could serve as an alternative method for evaluating mtDNA copy numbers and highlighting the potential of mtDNA copy number as a biomarker for glioblastoma development.^20^ Additionally, the evaluation of mtDNA content in multiple cancers has shown vast fluctuation, suggesting that mitochondrial copies are not particularly in stringent regulation.^21,22^ Certainly, the amount of mtDNA copy number may vary depending on tissue types and is mostly present mostly in high-energy cells such as skeletal, cardiac muscles, and brain cells.^3^

In theory, cancer cells display degradation of mtDNA, characterized by a significant buildup of oxidative stress, suggesting heightened glycolytic activity and insufficient adenosine triphosphate (ATP) production.^23^ The irregular mtDNA genome may lead to inefficient oxidative phosphorylation, resulting in elevated ROS levels and reduced ATP synthesis rates. As a result, it is hypothesized that elevated mtDNA content represents a compensatory response to mitochondrial respiratory deficiencies and mtDNA damage.^24^ Earlier research indicated a notable decrease in ATP synthesis within oncocytic thyroid tumors exhibiting elevated mitochondrial numbers and mtDNA content.^25^ Similarly, a recent study unveiled reduced ATP levels in mice displaying depressive-like behavior, alongside heightened mtDNA copy numbers.^26^

In this present study, variations in relative mtDNA content between cancerous tissues and blood controls were prominent among female patients and younger patients of age <40. Moreover, increased mtDNA content was more evident in grade I and II and non-glial tissues of brain tumors. However, our results noted that there were no significant associations detected between increased mtDNA copy numbers and those clinicopathological characteristics in cancerous brain tumor tissues. A previously published study also uncovered that there were no statistical differences between increased mtDNA copy number with clinical parameters in patients with glioma cases.^17^ The inability to clarify these connections could be due to the study’s small sample size across various tumor types, despite the significant difference in mtDNA content between cancerous tissues and blood samples. Thus, the increased sample size is warranted in determining the association between mtDNA content and clinical features in brain tumors.

Considering the significant variations of mtDNA copy number in brain tumor patients, we further examined the associations between the variable mtDNA content and the survival of the patients. Our findings confirmed that higher mtDNA content was linked to longer overall survival compared to lower mtDNA content among the patients. This observation aligns with previous studies that highlighted the correlation between increased mtDNA copy numbers and improved overall survival in GBM patients.^14,15^ Additionally, our observations revealed that elevated mtDNA content displayed a favorable prognosis in high-grade tumor patients. A prior study similarly emphasized the significance of the increased mtDNA content in the survival of high-grade glioma patients.^13^ Likewise, an intriguing investigation conducted by Sravya et al^11^ unveiled an inverse relationship between low mtDNA content and the survival rates of high-grade tumor cases. Consequently, it was hypothesized that an elevated mtDNA copy number could potentially contribute to the clinical outcomes observed in patients with brain tumors.

In the present finding, we detected a deletion of nucleotide A in *TFAM* sequences in a GBM patient (1 out of 41). The patient carried a single deletion with no amino acid changes and showed an increased mtDNA content level, indicating that there is no significant impact of those changes in the regulation of mtDNA content. However, a previous study revealed a high level of *TFAM* truncating mutation in 100% of cell lines and 74.4% of tissue samples of colorectal cancer, and the results also demonstrated mutated *TFAM* samples with decreased mtDNA content and mitochondrial instability.^5^ Another salient study found that heterozygous *TFAM* mutation reduces mtDNA copy number by up to 40% in vivo, while the homozygous mutation is embryonically lethal.^27^ Recently, reduced *TFAM* protein expression level was significantly associated with decreased mtDNA content and serves as a poor prognosis variable in non-small cell lung cancer.^28^

It is acknowledged that driver mutations contributing to tumor advancement are strongly linked to irregularities in mtDNA copy numbers. Previous studies highlighted that the expression of metabolic genes within mtDNA and the occurrence of somatic mutations expedite impaired mitochondrial functions by affecting mtDNA content.^5^ Nevertheless, it was stated that the alterations of several proteins might not necessarily influence the mtDNA replication process.^29^ An initial investigation revealed a slower rate of *TFAM* recovery compared to the mtDNA copy number in cells that had their mtDNA replenished with ethidium bromide, indicating that a higher level of mtDNA content might not rely heavily on increased *TFAM* levels.^30^ Nonetheless, *TFAM* comprises various functional domains that primarily control mtDNA content by engaging in specific and nonspecific sequence interactions within the mtDNA, ensuring the integrity of mitochondrial respiratory functions. Consequently, post-translational modifications influencing the turnover or stability of the TFAM protein can significantly impact the regulation of mtDNA content.^31^

In addition, we also examined large-scale deletions of mtDNA by the long-range PCR method that has been done in many functional studies.^10,32^ Our outcomes uncovered that none of the patients exhibit those deletions and were in line with a previous study by Danda et al^10^ which showed no large deletions found in patients with fibromyalgia syndrome. Nonetheless, an earlier study discovered a 12.2% of 8.7 kb deletion and 2.2% of ~5 kb deletion occurred in blood specimens of colorectal cancer.^33^ A different prior report detected a high prevalence of large mtDNA deletions (3938 and 4388 bp) in cancerous tissues compared to the non-cancerous counterparts of breast cancer.^34^

While large deletions in mtDNA have traditionally been traditionally viewed as infrequent occurrences (sporadic events) in comparison to point mutations, their substantial impact becomes more pronounced in aging, mitochondrial diseases, and cancers.^35^ This disparity likely arises from large deletions within the mtDNA, resulting in the loss or truncation of multiple structural genes responsible for encoding mitochondrial respiratory enzyme subunits.^33^ Moreover, the susceptibility of the mtDNA genome to damage and the presence of an impaired repair system might result in the prolonged persistence of mutated and deleted species within the cell, rather than undergoing proper repair. This scenario could potentially elevate oxidative stress, consequently fostering the development of cancer.

Limitations, drawbacks, or shortcomings: Con­siderations should be given to the limitations of our study. First, despite incorporating all available data, the sample size remains small. A smaller sample size can introduce biases and random errors, necessitating a larger study to thoroughly explore potential connections between mtDNA irregularities and clinical traits in these patients. Second, mtDNA copy numbers for the normal control were measured from whole blood DNA, chosen due to its easy accessibility. Non-tumor tissues near the brain tumor were not used as controls because high-grade tumors grow quickly, invade, and damage surrounding normal brain tissues, making them challenging to use as controls without harming nearby healthy tissues. Alternatively, the patient’s own peripheral blood was used as a control. Third, variations in age, sex, and phenotypes among study groups might impact the diversity observed in these associations. For instance, the prevalence of mtDNA content in this study could be influenced by older age cases, as mtDNA copy number tends to be lower in aged populations. Conclusively, addressing these needs entails an expanded sample size and a meticulously planned research framework to comprehensively evaluate the connection between mtDNA content variations and clinical characteristics.

In conclusion, this study represents the first investigation in Malaysia exploring the correlation between variations in mtDNA copy numbers and the occurrence of brain tumors. The findings highlight elevated mtDNA copy counts in brain tumor tissues in contrast to controls, indicating possible significance within this cancer context and hinting at improved survival rates among brain tumor patients. Furthermore, future research should delve into the detailed mechanisms and potential roles underlying mitochondrial genome alterations and copy number variations in tumor progression.

## Figures and Tables

**Table 1.** Clinicopathological Features of Brain Tumor Cases

Parameters	Number of patients (%)
Gender	3
Male	23 (56.1)
Female	18 (43.9)
Age (years)	3
<40	18 (43.9)
≥40	23 (56.1)
Mean	41.9
Range	5-73
Tumor grade	3
Low grade (I and II)	25 (61.0)
High grade (III and IV)	16 (39.0)
Histological types	3
Glial tumors	24 (58.5)
Gemistocytic astrocytoma II	3
Oligodendroglioma III	4
Ependymoma II	3
Anaplastic pleomorphic xanthoastrocytoma III	4
Glioblastoma multiforme IV	5
Schwannoma I	5
Non-glial tumors	17 (41.5)
Medulloblastoma IV	3
Meningioma I	11
Meningioma II	3
Survival status	3
Censored	27 (65.9)
Relapse	14 (34.1)

**Table 2.** Correlation of mtDNA Content in Cancerous Tissues and Paired Controls

	Cancerous	Blood	Fold	*P*
∆CT = Ct_mtDNA_ – Ct_nDNA _3	7.35	11.81	∆∆CT	<.001^a^3
3	(5.08)	(2.37)	7.35 − 11.81 = −4.5	3
3	3	3	2^−∆∆CT^ = 22.6	3
Relative mtDNA content	0.0123	0.0006	3	<.001^a^3
= 2 × 2^-∆CT^3	(0.04)	(0.001182)	3	3
Increased mtDNA content	33	8	3	<.001^c^3
Correlation (Ct_nDNA_ : Ct_mtDNA_)	0.248^b^3	0.039^b^3	3	3

Assumption of normality is fulfilled. IQR values are shown in the parentheses.

^a^Mann–Whitney *U*-test.

^b^Pearson correlation test.

^c^Chi-square difference test.

**Table 3.** Data of Brain Tumor Patients and the Relationships Between Increased mtDNA Content in Brain Tumor Tissues and Clinicopathological Parameters

Parameters	Group	Cases (%)	mtDNA Content^a^	*P* ^b^
Gender	Male	23 (56.1)	0.0063 (0.03)	.379
	Female	18 (43.9)	0.0186 (0.04)	3
Age (years)	<40	18 (43.9)	0.0115 (0.04)	.331
	≥40	23 (56.1)	0.0123 (0.04)	3
Tumor grade	Low grade (I and II)	25 (61.0)	0.0154 (0.04)	.570
	High grade (III and IV)	16 (39.0)	0.0078 (0.05)	3
Histological types	Glial-tumors	24 (58.5)	0.0087 (0.03)	.534
	Non-glial tumors	17 (41.5)	0.0203 (0.04)	3

The non-normality assumption is fulfilled.

^a^Median (IQR).

^b^Mann–Whitney *U-*test.

**Table 4.** Previous Studies of mtDNA Content in Glioma Cohort

Study Type	No. of Cases	No of Controls	Method of mtDNA Quantification	mtDNA Gene	nDNA Gene	Main Outcomes	Study, Year (Reference)
Case–control study	10 GBM patients with histologic oncocytic features	18 primary GBM patients	Real-time PCR	*MT-ND2*3	*FALSG*3	9/10 had markedly increased mtDNA content compared to controls. Immunohistochemical results shown consistent association with mtDNA copy number. 3	Marucci et al, 2013^16^3
Case–control study	414 peripheral blood lymphocytes of glioma patients	414 healthy controls	Real-time PCR	*MT-ND1*3	*HGB*3	Glioma patients demonstrated increased mtDNA copy number compared to controls and significantly associated with the increased risk of glioma 3	Zhang et al, 2014^17^3
Comparative study	336 blood specimens of glioma patients	N/A	Real-time PCR	*MT-ND1*3	*HGB*3	Increased mtDNA content significantly associated with poor prognosis in patients with early age, high-grade glioma, and radio chemotherapy 3	Chen et al, 2016^12^3
Case–control study	124 cancerous glioma tissues	124 non-cancerous glioma tissues	Real-time PCR	*MT-ND1*3	*ACTB*3	Increased mtDNA content displayed in glioma patients and significantly associated with seizures. Decreased mtDNA content shown in recurrent cases than those in non-recurrent cases. 3	Zhang et al, 2015^13^3
Case–control study	395 peripheral blood of glioma patients	425 blood of healthy controls	Real-time PCR	*MT-ND1*3	*HGB*3	Increased mtDNA copy number compared to healthy controls and this increase was associated with an increased risk of glioma GBM and high-grade gliomas had significantly reduced mtDNA copy number compared to their counterparts in newly diagnosed cases. 3	Shen et al, 2016^19^3
Comparative study	67 primary GBM patients	N/A	Real-time PCR	N/A	N/A	Increased mtDNA copy number was markedly correlated with better overall survival in young adult GBM patients. 3	Dardaud et al, 2019^15^3
Retrospective study	130 newly diagnosed GBM patients and 32 recurrent GBM patients	35 non-cancerous brain tissues (FFPE)	Real-time PCR	*MT-ND1*3	*RNase P*3	Reduced mtDNA content in GBM patients was associated with poor overall survival, progression-free survival, and wild-type IDH. Increased mtDNA copy number was found in recurrent GBM patients who received post-radiation therapy compared to newly diagnosed cases. 3	Sravya et al, 2020^11^3
Case–control study	35 DIPGs and 25 supratentorial HGGs tissues	19 normal brain tissues	Real-time PCR	*D˗loop *and* MT-CO2*3	*ACTB*3	Reduced mtDNA content in pHGGs correlated with higher cell migration and invasion, therapeutic resistance, and in vivo tumorigenicity. 3	Shen et al, 2020^18^3
Comparative study	232 primary GBM tissues	N/A	Real-time PCR	*MT-CO1 *and *MT-ND4*3	*B2M *and* GAPDH*3	Overall survival was significantly longer in the high mtDNA level vs. low mtDNA level subgroup in younger patients and longer in the low mtDNA level vs. high mtDNA level in older GBM patients. 3	Sourty et al, 2022^14^3
Comparative study	44 GBM patients	44 control individuals	Real-time PCR	*MT-ND1 *and *MT-ND5*3	*SLCO2B1 *and *SERPINA 1*3	Decreased mtDNA content in exosome and brain tissues of tumor samples than those in the controls of GBM patients. 3	Soltész et al, 2022^20^3

*ACTB*, β‑actin; *B2M*, β‑2‑microglobulin; DIPGs, diffuse intrinsic pontine gliomas; *D‑loop*, displacement loop; *FASLG*, Fas ligand; *GAPDH*, glyceraldehyde‑3‑phosphate dehydrogenase; GBM, glioblastoma; *HGB*, human globulin; *MT-CO1*, mitochondrially encoded cytochrome c oxidase I; *MT-CO2*, mitochondrially encoded cytochrome c oxidase II; *MT‑ND1*, mitochondrially encoded NADH dehydrogenase 1; *MT‑ND2*, mitochondrially encoded NADH dehydrogenase 2; *MT-ND4*, mitochondrially encoded NADH dehydrogenase 4; *MT-ND5*, mitochondrially encoded NADH dehydrogenase 5; pHGGs, pediatric high-grade gliomas; *RNase P*, Ribonuclease P; *SERPINA 1*, serpin family A member 1; *SLCO2B1*, solute carrier organic anion transporter family member 2B1.
